# A Series of Cases Illustrative of Some New Views upon the Pathology and Treatment of Epidemic Dysentery

**Published:** 1845-10

**Authors:** O. Bailey

**Affiliations:** Lancaster Co., Pennsylvania


### MEDICAL EXAMINER,

AND

### RECORD OF MEDICAL SCIENCE.

NEW SERIES—No. X.—OCTOBER, 1845.

### ORIGINAL COMMUNICATIONS.

*A series of Cases illustrative of some new views upon the Pathology and Treatment of Epidemic Dysentery.* By O. Bailey, M. D., of Lancaster Co., Pennsylvania.

The ordinarily fatal character of this disease throughout this section of country, under the common course of treatment, and the frequency with which it is preceded by, alternated with, or followed by acute rheumatism, has induced the writer to adopt some peculiar views with regard to it. From these he has been led to apply a corresponding treatment, which having been to a great degree successful with himself and his professional friends around, determines him to offer it to the consideration of the profession, and the test of a more extensive experience.

Many circumstances appear as evidence that epidemic dysentery, like acute rheumatism, is the result of spinal irritation, the termination of which in inflammation being the cause of its fatality. To this spinal irritation in dysentery, is added a peculiar dry harsh condition of skin, which probably determines the abdominal character of the affection, and adds to the rapid development of the irritation. Under such conditions, the antiphlogistic and revulsive agency of cupping over the spine, and the action of some diaphoretic, would be the appropriate means to be used in the treatment. The following notes exhibit some comparative results—comparing this with the methods commonly pursued in the treatment.

Of 42 cases treated in the ordinary manner, (by calomel, opium and ipecac.) occurring in the vicinity of Andrew's Bridge, during the summer and autumn of 1842-3, 18 died and 24 recovered.

| | | | |
|---|---|---|---|
| Say | 23 adults. | 8 of whom died, | 15 recovered. |
| | 19 under 10 years, | 10 " " | 9 " |
| | 42 | 18 | 24 |

Average time in those dying, $8\frac{1}{3}$ days.

" " recovering, 13 days.

In 1844, between July 28 and Aug. 10, there first occurred 5 cases, which were treated in a similar manner; 3 were under, and 2 over 10 years of age; 4 died and one recovered. Average time in those dying, $6\frac{1}{2}$ days; the single recovery taking place in 7 days. Remarkable emaciation, with rigidity and contraction of the abdomen in those who died.

The 6th case occurring under the writer's notice during this year, was the father of three of those who died. The same symptoms were observed, and similar treatment pursued for three days without any apparent advantage, the patient being more debilitated, with a contracted and rigid abdomen. The injections of warm water which had been recommended were abandoned, in consequence of the irritability of the rectum.

On the fourth day, at 11 o'clock, 6 cups, (that being as many as could be borne,) were applied near the spine, and an infusion of thoroughwort (Eupatorium perfoliatum,) was directed to be given freely.

At 7 o'clock P.M., the patient had been up only three times since the cupping, and the discharges were now more like those of ordinary diarrhœa. Complained of soreness over the abdomen. The cups were reapplied over the same scarifications, the blood flowing more freely than at first, and the infusion to be continued, adding warm water injections, which can now be borne.

Fifth day, at 10 o'clock. Had been up but once since last visit, and complained only of soreness over the abdomen, with pain and slight tumefaction of the left wrist. Applied three cups over the origin of the brachial nerves. This relieved the wrist, and the soreness of the abdomen passed off after a short time.

Case 7th.—Aug. 17th. Mr. C., a friend and pupil of the writer, who had accompanied him in his visits to the above mentioned patients, was taken in the morning with symptoms of the disease. These increased towards evening, the pain and tenesmus being severe, and the calls to stool frequent. At this time he was seized with a chill, followed by uneasiness in the back and limbs.

Determined in this case to abandon entirely the use of opium and purgatives, as these appeared to have otherwise than a beneficial effect.

Jugs of warm water were applied to the feet, and the infusion of the eupatorium given as warm as it could be taken. In a few minutes all chilliness had disappeared and a free perspiration established. The administration of fifteen grains of ipecac. now produced free emesis, and a slight remission of the distressing symptoms. At 12 o'clock at night, however, the patient was much worse than in the evening, the tenesmus and calls to stool having increased, and being accompanied by considerable fever.

Venesection to f℥xij.

In the morning of the second day, there being no abatement of symptoms, 6 or 7 cups were applied over the spine, extending from the inferior cervical vertebra to the sacrum, and taking 5 or 6 oz. of blood. The infusion was again resumed. Subsequent to this there was but one dysenteric discharge. In five or six hours after the cupping an enemata of tepid water was administered. This produced a free evacuation of the bowels, after which all traces of dysentery had disappeared.

Case 8th.—Mrs. R., mother of one of the children mentioned as having died. Was attacked in a milder manner,—refused to submit to cupping, and was therefore treated by injections and the infusion of eupatorium. Recovery in twenty days.

Case 9th.—Aug. 20. Mr. R. Visited 12 hours after the attack, by the writer's son, Dr. E. J. Bailey. Recovered after the first application of cups.

Case 10th.—Oct. 1st. Seen also by Dr. E. J. Bailey, on the fourth day after the attack. The termina and tenesmus were relieved by the first application of cups, leaving only a soreness of the abdomen, with a mild diarrhœa for two or three days.

Case 11th.—Miss C., a lady of delicate constitution, was attacked with pain in the back, followed in a short time by pain, redness and swelling of the ankles, knees, wrists and elbows in succession. For this she was bled from the arm to f℥x. and had ten cups applied over the spine. From this treatment she experienced relief in a great measure, the bowels being open, and the skin covered with perspiration. This improved condition was continued until the second day after the treatment, without any thing further being done. On the morning of the third day, after having passed a restless night in consequence of pains in the lower extremities, she became easier, and fell asleep in her chair. Being suddenly aroused by a noise, she felt the pains much aggravated, accompanied by a prickling or tingling sensation in the extremities. These subsided in half an hour, and left her quite free from pain. She then, for the first time, experienced an uneasy sensation in her bowels, with an inclination to remain longer than usual at stool. Later in the day, the tormina and tenesmus were constant and distressing, the bloody mucus coming away in drops.

Ten cups were applied in the region of the spine, commencing at the inferior cervical vertebra and extending to the sacrum, nine or ten ounces of blood being taken by them. The infusion of thoroughwort was given freely, as warm as it could be taken, and an injection of tepid water. After these she remained quiet until the next morning, when her bowels were moved gently, and not a trace of dysentery remained.

To these the following case of singular changing may be added.

Mr. D., æt. 60, was attacked by pleuritis on the 24th of December, confining him to bed for six or seven days. When he had so far recovered as to sit up and walk about his chamber, he felt an uneasy sensation in the region of his heart, accompanied by dyspnœa and intermittent palpitations. These in a short time were followed by a numb pricking sensation and loss of motion in the right arm and leg, with slight tumefaction and pain in the ankle and wrist. These again were succeeded by small and frequent discharges of bloody mucus from the bowels. These had previously been in a free and healthy condition, and no drastic cathartics had been used.

The particulars of the following cases have been kindly furnished to the writer by his friend, Dr. A. V. B. Orr, of Cooperville, having been treated in accordance with the new views upon the subject.

Case 12th.—Aug. 22, 1844. Was called, says Dr. Orr, to visit E. H.; æt. 20; miller by trade. Had been attacked two days previously, and presented at the time of visit the following symptoms: Pulse 100, full and bounding; tongue intensely red at the tip and edges, bearing a yellow fur in the centre; some nausea; constant pain in the abdomen, unusually severe, and increased whilst at stool, to which he was obliged to rise every 15 or 20 minutes. These stools were very fœtid, and were composed of blood and mucus, or small quantities of blood alone; patient could scarcely be induced to leave the close stool, and when in bed was very restless from the great pain. The skin was constantly harsh and dry. Upon examination of the spine, a slight degree of tenderness was detected about the usual place by *pretty firm pressure.* With some difficulty four cups were applied to the spine, taking four or five ounces of blood. The pain now abated, and in the course of an hour the skin became moist. Directed five grains of Dover's powder to be given every third hour, with an ounce of olive oil, and twenty drops of tincture of opium at bed time.

On the 4th day, found the patient had rested tolerably well; pulse not so frequent, and softer; tongue less coated; no return of pain since the cupping; only three stools since, with but little blood; skin continues moist; soreness over the abdomen.

Directed fomentations to the abdomen, with olive oil at bed time as before, omitting the powders.

On the 5th day, found the patient sitting up. Tongue cleaning; slight tenderness over the abdomen, with gentle diarrhœa. This latter continued for a few days, after which the patient attended to his business as usual.

I feel perfectly safe in saying that I have seen several cases where stronger men, labouring, apparently, under less severe forms of the disease, have died in eight days under the usual treatment.

Case 13th.—Aug. 28. Was called to visit J. C., æt. 40; found he had laboured under dysentery for four days. Pulse 90, small and irritable; tongue clean and glazed; stools frequent and scanty, consisting principally of blood; pain constant, but not so severe as in the last case; great prostration and emaciation, with giddiness and a tendency to syncope whilst on the close stool.

Applied four cups to the spine, with fomentations to the abdomen, and gave an enema of one pint of starch with half drachm of tincture of opium.

On the 6th day of the attack, found the symptoms much abated. Directed two additional cups to the spine, with a powder consisting of five grains of Dover's powder with one grain of acetate of lead, to be given every third hour for 12 hours, and half an ounce of olive oil at bed time, continuing the fomentations.

On the 7th day, patient had passed a good night. Pulse soft and more full; tongue moist; pain slight; had but three stools since last visit; skin moist through the night; still some soreness over the abdomen.

Directed mustard cataplasm to the abdomen, and olive oil at bed time as before.

The patient recovered rapidly without further treatment, except the olive oil, which was continued for a few evenings.

In this case, as indeed in all the cases wherein I have used the cups, the pain has abated immediately, and never again returned with the same intensity; sometimes, however, requiring to be repeated in cases when the disease had been of some days standing.

Case 14th.—Was called to G. W., æt 20. Found he had been under Thompsonian treatment for two or three days, and that the disease, from an ordinary diarrhœa, had degenerated into dysentery, probably from the irritating nature of the treatment used. Pulse 90, full and tolerably hard; tongue clean, but very red; skin dry; pain severe; stools frequent, consisting of small quantities of bloody mucus.

Six cups were applied to the spine, and an ounce of olive oil directed to be given at bed time.

The following day he was sitting up, and a repetition of the olive oil was alone necessary to perfect the cure.

I feel perfectly satisfied that there is more mischief done in dysentery by cathartics than in almost any other disease. I have found the *very mildest* alone admissible, and indeed, have frequently seen mischief arise from the use even of castor oil. From these reasons I have of late used the olive alone, and find it preferable to any other.

Case 15th.—Sept. 8th. Was called to W. H., æt. 15. Had been sick since the preceding day, but was still able to sit up. Pulse nearly natural; tongue red at the tip and edges, covered in the centre with a white fur; stools hourly, accompanied with pain, the matters voided being watery and tinged with blood; skin dry; breath offensive. Concluded to omit the cupping and watch the result. Directed three powders, each consisting of two grains of calomel and two of ipecac., with one grain of opium, to be given at intervals of four hours, and followed by half an ounce of olive oil.

On the 3d day of the attack, found the patient much as before, the stools being, however, less watery. Directed a continuance of the treatment.

On the 4th day. Pulse accelerated; tongue clean but very red; skin moist; stools as frequent but contain more blood, mixed with bilious matters, and very offensive; abdomen tender on pressure.

Substitute powders of five grains of Dover's powder with half grain of acetate of lead every third hour, for those before used; apply mustard to the abdomen and repeat the olive oil as before. This treatment was continued, without much change in the symptoms, until the morning of the sixth day, when I was called to visit the patient in haste. Found the symptoms of dysentery all gone, and in their stead a well marked case of rheumatism, the ankles and knees being swelled, red, and exquisitely painful, the legs being flexed on the thighs, and the patient unable to extend them without producing the greatest pain. The sensibility was so great that he could not bear the clothes to be moved.

Applied six cups to the spine, and directed infusion of thoroughwort, with sulphur as a laxative.

On the 7th day, some abatement in the pain, but still unable to extend the legs.

Applied four additional cups, and directed the infusion continued.

On the 8th day, no pain in the joints, but much stiffness in the knees. Three cups to the spine, continuing the infusion and repeating the sulphur.

During the night the patient was seized with diarrhœa, which continued for a few days, no further inconvenience from the rheumatism being experienced, except a slight stiffness in the joints.

From the first accession of dysentery until the return to health, this patient never left his room, so that there was here a perfect transition from dysentery to rheumatism, without any appreciable cause, the two being quite distinct and each well marked.

The following case, although not one in which the cups were used, may serve to illustrate the pathological relations between dysentery and rheumatism.

March 20th, 1842. Was called to A. B., æt. 16. Complained of pain in the right knee with slight stiffness. Pulse 100, very hard; tongue slightly furred; bowels costive; skin dry. Has not received any injury of the part, but had been very wet from exposure during a shower two days before. Has had more or less pain in the part since, growing gradually worse.

Venesection from a large orifice to f℥x., the blood being buffy and cupped. The pulse became softer, but the pain was not affected by this bleeding.

Directed to take ten grains each of calomel and jalap, and to rub the part with a liniment of ammonia and tincture of opium. On the fourth day found the patient had slept more during the night, and that the bowels had been moved twice, but that the pain in the knee had increased, and some tumefaction had occurred, with slight redness extending above the joint. Pulse 90, compressible. Directed a blister to the joint, and a powder of two grains each of calomel and ipecac., with one and a quarter grains of opium, to be given every three hours for nine hours, to be followed by half an ounce of castor oil, at bed time.

On the 5th day, found the patient worse, the arm of the same side being now affected. Directed a consultation with my friend Dr. M. at four o'clock. At this time found the extremities of the other side implicated, and the skin covered with a cold moisture.

Determined to try a gentle ptyalism; and directed calomel, ipecac. and opium, every two hours, until this should supervene; with the application of mustard to the parts.

On the 6th day, found the gums affected, and therefore discontinued the calomel, etc.,—but little abatement of symptoms. Ordered half an ounce of castor oil, and the parts to be bathed with warm brandy.

On the 7th day, found the pain had been entirely translated to the bowels, the abdomen being very tender. Stools frequent, consisting of bloody mucus.

Directed a large blister to the abdomen, with enemata of starch and laudanum, to be repeated as occasion may require.

On the 8th day, found the blister and other measures had afforded no relief.

On the 10th day, the patient died, with all the symptoms of dysentery.

In this case the periods of disease were quite distinct, and the translation immediate, without apparent cause.

Case 16th.—I have now under my care, a patient æt. 18, in whom this translation took place several times before the cups were used. Four cups were at length applied to the spine, since which he is rapidly recovering.

I might give you several cases in which I believe I have cut off attacks of dysentery in 24 hours, but as there might be some doubt as to the true nature of the disease in these early stages, I purposely withhold them.

I was called during the past summer to visit J. P., æt. 60. He had laboured under dysentery for two or three days. Under the usual treatment, in a few days he appeared to get better, and no doubt of his final recovery was entertained, as the case was rather a mild one. He was, however, suddenly seized during the night with dyspnœa, palpitation of the heart, etc., the dysenteric symptoms immediately ceasing. His death occurred during the following day.

The subjoined communications from two other of the writer's esteemed friends, have been kindly sent him in answer to some inquiries relative to the disease as it has occurred in their practice.

*February*, 1845.

*Dear Doctor*,—Your enquiries with regard to dysentery, I take pleasure in answering, etc. It occurred to a considerable extent in my practice of last summer. The treatment which I employed in the main, consisted in bleeding, calomel and opium, followed, when much pain and tenderness continued, by cups over the abdomen, in conjunction with such other remedial measures as are commonly employed by the profession. In a number of cases my attention was attracted to a painful and swelled state of the joints, occurring some four or five days after the disease gave way. I readily concluded that these cases were rheumatism, and, when they became subjects of attention, treated them as such. In several cases a most troublesome degree of tenesmus remained after the disease appeared relieved. These I found exceedingly difficult to manage. Temporary relief could always be obtained by some anodyne enemata ; yet it was prone to return. Conversing with a friend upon the subject of the prevailing disease, he mentioned to me your recent views in regard to the primary cause of dysentery. The following day I determined, in accordance with those views, to try the effect of cupping over the lumbar region, in order to relieve this distressing tenesmus. Three cases presented themselves, and the result was a prompt and effectual cure. I was sorry your important opinions in reference to this disease had not come to my knowledge at an earlier date, as my opportunities for testing the efficacy of a treatment, which, in accordance with your theory, would seem most appropriate, were very numerous. The ordinary duration of dysentery was from 10 to 14 days; at least so far as regards those cases which came under my own observation.

With much esteem, I remain, yours truly,

D. H. Agnew.

To Dr. O. Bailey.

*Oxford, February,* 1845.

*Dear Sir,*—In reply to your inquiries respecting dysentery, I would state that during the summer and autumn of 1843, I had over 125 cases, many of which were of a decidedly congestive character. A number of these patients, as soon as they became convalescent, were seized with rheumatism, and many others complained of stiffness of the joints.

During the summer and autumn I had but 35 cases ; 20 of these were affected as above by rheumatism. Some of those who had stiff joints accused me of having given them too much mercury, although ptyalism had not occurred. As, however, it passed off in three or four weeks at most, and they were not afterwards troubled by it, they became satisfied that it was not the effect of the medicine. Ptyalism was not excited in one of these cases.

Yours, etc., D. W. Hutchison.

To Obed Bailey, M. D.

Tabular view of the cases above referred to comparative of the peculiar treatments used.

47 cases treated by calomel, etc., 22 died, 25 recovered.
10 cases treated by cupping, etc., all recovered.

